# An Essay on the Use of Narcotics and Other Remedial Agents, Calculated to Produce Sleep, in the Treatment of Insanity

**Published:** 1846-07

**Authors:** 


### Art. V.

1. *An Essay on the Use of Narcotics and other remedial agents, calculated to produce Sleep in the Treatment of Insanity.* By Joseph Williams, m.d.—*London,* 1845. 8vo, pp. 120.

2. *Practical Notes on Insanity.* By John Burdett Steward, m.d.—*London,* 1845. 8vo, pp. 120.

I. To the essay of Dr. Williams was awarded a premium placed at the disposal of the King and Queen's College of Physicians, by the Lord Chancellor of Ireland, for an essay on some subjects connected with the treatment of Insanity.

The author commences his essay by alluding to the very varied causes producing the insomnolence which so frequently precedes an attack of insanity, and forms, in general, so important a feature of the disease throughout its progress; and the consequent necessity, in a work treating on the means of procuring sleep in insanity, of discussing remedies which cannot be regarded as narcotics. Accordingly, we have in his essay articles on bleeding, general and local, purgatives and emetics, narcotics, stimulants and tonics, warm baths, exercise, &c.

After a brief allusion to the several forms of the disease and its varieties, as adopted by systematic writers, together with an outline of the causes most frequently conducing to its development, he refers to the importance, at the commencement of an attack of insanity, of accurately discriminating its cause, and the state of system under which it appears, as upon the treatment pursued at this period most frequently depends the result of the case, and whether it shall rapidly subside or terminate in a chronic form of the disease.

"There is always extreme irritability in incipient insanity. Generally the brain suffers first, then some other organ. The great object, however, is always in the first to allay irritation, and to endeavour to ascertain whether the brain was primarily affected, or whether insanity followed some visceral affection. It is of the greatest importance to determine whether insanity is symptomatic or idiopathic; whether the result of mere error of perception, [?] or whether the medium through which we reason is at fault. Mind is independent of matter [!] and it may by some sudden shock become incapable of perceiving, discriminating, or judging correctly; and it is in such cases when tranquillity has been restored by narcotics, that the metaphysical treatment has been successful. If the excitement consequent on reaction in these cases be not speedily lulled, the brain itself often becomes congested or inflamed; and this continuing, symptoms increase, and those alterations in the brain and membranes so frequently observed more or less speedily occur." (p. 28.)

*Bleeding.* Dr. Williams agrees with most writers on this subject, in recommending caution in the use of general bleeding, which he regards as applicable only to the cases of the disease appearing in strong, robust persons, living in country situations, and in whom the attack has followed the suppression of accustomed evacuations, as epistaxis in men, or the suppression or cessation of the catamenia in females. In these cases, also, it is rarely, if ever, applicable, except in the early stage of the disease, and must be employed sparingly. In ordinary cases he prefers local to general depletion; the abstraction of blood by the cupping-glass, or by leeches, being less likely to produce undue depression afterwards, or to be followed by reaction. He alludes to what we have frequently observed, the soothing effect produced on maniacal patients by the gradual abstraction of blood by leeches, and regards this mode of treatment as not having received sufficient attention. In all cases when antiphlogistic measures are about to be had recourse to, it becomes of the greatest importance to discriminate between the symptoms resulting from active inflammatory action and those originating in irritation; and in many cases where general or local depletion is required, it will be necessary carefully to support the strength of the patient, or even to exhibit stimulants at the same time.

"Many cases of insanity arise from extreme irritability dependent on prostrated powers; and to support the person by good nutritious food, and sometimes even with brandy and wine, at the same time soothing the system by procuring refreshing sleep at night by morphia, will speedily evidence the advantages of such treatment. The great error originally was allowing the powers to sink; it is of the greatest importance that those powers should be supported; the nervous excitation must be calmed. In these cases mistakes are but too frequently made; irritation is confounded with inflammation. The maxims so ably taught by Mr. Travers are forgotten; the object being to calm the action, not to diminish from the power—this nervous power being much more easily depressed than raised. Should this advice be neglected, and bleeding be ordered, stupor, or coma, or confirmed mania may be the consequence. In many cases, when there is the most ferocious delirium with great muscular power, yet the pulse is very quick, weak, and fluttering, and even the slightest depletion at once knocks down the powers; but even if the patient should again rally, there is great danger of his becoming idiotic." (p. 32.)

Purgatives, antimonials, and emetics are applicable in conjunction with the general antiphlogistic treatment, but more especially may be employed in those cases of inflammatory excitement when depletion has already been carried as far as may be regarded as safe, or when the more active antiphlogistic measures are inadmissible.

"Many cases of vigilantia, dependent on monomania, or even furious mania, will yield to ant. potass. tart., and often, on the vomiting ceasing, refreshing sleep will follow. It has been remarked by Dr. Cox that one third the usual dose of tartar emetic will prove efficient if a narcotic has been given the night before; generally, however, full doses are required. . . . . . The continued action of tartar emetic cannot be too much lauded in some incipient cases of mania; while under the influence of antimony, the patient seems rational; it is withdrawn, reaction occurs; the eyes roll or remain fixed, noise succeeds tranquillity, the head again becomes hot. As evening sets in symptoms increase, and the patient, with unclosed eyes, passes a restless and boisterous night; whereas, had the action of antimony been kept up, placidity, if not actual sleep, would have been substituted for extreme restlessness and violence." (p. 45.)

*Opium.* "Opium is contraindicated when there is great heat of skin with extreme restlessness, and determination of blood to the head; and all authorities seem agreed that it should never be administered when the system is plethoric, unless depletion or purgation, or both, have preceded it." (p. 47.)

"Delicate and debilitated constitutions, with spasmodic irritability, generally bear opium well, and this perhaps accounts for its less frequently disagreeing with females than with males. When the nervous system is the most highly developed opium is often the most useful, and is especially indicated in those cases of vigilantia and restless cases resulting from nervousness. In puerpural mania, when it has been necessary to deplete or purge, large doses of opium are doubly necessary; and should sleep follow, the attack will generally be alleviated or suspended. Opium is essentially indicated when the system is depressed, when it often acts as a charm; and by its stimulating properties is far more useful than Battley's sedative, or the preparations of morphia." (p. 52.)

Opium is generally very useful in suicidal cases, or those resulting from continued intoxication, and after loss of blood; or when the person is much exsanguined and depressed, and has a dry, cold skin. Generally it will be well to give it in a full dose (from two to five grains). Dr. Burrows begins with three grains, and repeats one grain every two or three hours, never allowing the quantity given to exceed twelve grains. In cases where there is febrile excitement, it may be combined with calomel, antimony, or James's powder. It may also be given in combination with camphor, hyoscyamus, or digitalis, or in the form of pulv. cretæ co. c. opio, and may be applied by enema or injections. If the purely sedative effect be desired, the preparations of morphia, or the sedative liquor, may be substituted for the opium.

*Digitalis.* "When there is considerable arterial action with vigilantia, after being well purged, digitalis will very often reduce the power and frequency of the pulse, and sleep follows. In mania, with or without henbane or camphor, digitalis will be found a most useful narcotic, but if the excitement be excessive, and this arise from acute inflammation, tartarized antimony must precede it." (p. 67.)

The tincture is the most eligible form for exhibition, and may be given in doses of 10m. every six hours; if the pulse does not become less frequent in five or six days, it must be discontinued.

*Hyoscyamus.* "Hyoscyamus is especially useful in nervous habits, and is particularly indicated in monomania, and even the temporary quiet from it in mania is often of the greatest benefit. When there is excessive nervous irritability, it has often a remarkably calming and soothing effect; it may also be given when there is vascular excitement, when opium is strongly contraindicated; it does not excite the brain in these cases, and is often found to reduce arterial action. When patients awake after the sleep caused by henbane there is not that confusion of thought, that stupified expression, nor heat of skin, and dryness of the fauces and tongue, so often seen when other narcotics have been taken. . . . . When a sedative has to be continued from day to day, or several times during the day, hyoscyamus will often be the very best we can select; as, in addition to its tranquillizing effects, it will not check if it does not actually cause diaphoresis, while it promotes the flow of the urine, and also relaxes the bowels. It acts almost as a specific in some cases of monomania, causing tranquil sleep, and a quiet, placid waking." (p. 69.)

Hyoscyamus may be given in combination with opium or camphor, and other remedies, according to the circumstances. Of the extract five to ten grains may be given as a dose, and five grains may be repeated every four hours. Some prefer to give it in large doses at once, and in this way ten to fifteen or thirty grains may be given. It should never be exhibited as an enema, as fatal results have attended this practice.

*Warm baths.* "There are many cases of insanity where there is want of sleep, in which it would be perfectly useless to prescribe narcotics, but very good effects invariably follow the judicious employment of warm tepid baths. Much discrimination is necessary to determine as to the heat best adapted to the particular case, and the greatest care must be taken not to raise the temperature too high; it should never exceed 98° F., or it will act as an excitant, and may even induce apoplexy; 96° F. may be usually considered as the best temperature of a warm bath for the insane. Persons of a nervous temperament bear a higher temperature than the bilious; and the warm bath is more decidedly useful in cases of melancholia than in other forms of insanity; but it will be generally found a very powerful means of diminishing cerebral congestion, and allaying irritation in most maniacal cases. . . . It is often asked how long should a patient remain in the bath, and how frequently should it be repeated. It may be necessary to order a bath daily, or even twice a day, and the patient may be immersed half an hour, one hour, and even two hours, the time depending on the effect produced. . . . . The warm bath will be found very effectual when the circulation is sluggish, skin and feet cold, when often half an hour's immersion will ensure a good night's rest. The first effect produced is languor; as soon as this is perceived the patient must be removed, or actual syncope may occur." (p. 91.)

With these extracts we take our leave of Dr. Williams's essay. In a treatise of this kind we must not expect any very novel or original views. Dr. Williams has, however, bestowed great attention on his subject, and presents us, in a small compass, with a large amount of practical and judicious observations on the various remedies employed in the treatment of insanity. His metaphysics are of the old school, and his pathology is not of the newest; still we commend his little volume to the members of the profession, who, whether engaged in general practice, or in the more exclusive treatment of mental affections, will find it a useful compilation.

II. The notes of Dr. Steward have reference to the treatment of insanity generally, though the largest share of attention is devoted to the discussion of the moral management of the insane.

In the preface, and repeatedly throughout the course of his work, Dr. Steward alludes to the, as we think, now universally admitted necessity for confiding the care of the insane only to qualified medical men; and his remarks on the demand for a change in this particular appear more applicable to what was the state of matters five or ten years ago than to the practice which prevails at the present time. So far as our observation serves us there are now very few institutions, whether public or private, for the treatment of insanity, in which the superintendence is not vested in resident medical men. We are, indeed, far from thinking that much does not remain to be accomplished in the improvement of the system of treating these cases; but we believe that the change has already commenced, and is rapidly advancing. In proof of this we may refer to the attention which is at present paid in the selection of medical superintendents to our public institutions, to secure those who are not only regarded as possessed of a full share of medical knowledge, but also of an adequate experience in the management of establishments for the special treatment of insanity. We may also instance the facilities afforded by the managers of some of our public asylums for the acquirement, by the rising members of the profession, of a practical acquaintance with the treatment of insanity (as in the Lectures delivered by Dr. Conolly at Hanwell, and Dr. Sutherland at St. Luke's, and in the resident studentships at the Gloucester, Morningside, and Glasgow asylums) as affording proof that the enlightened portion of the public is already fully alive to the necessity of a practical study of those cases to medical men, whether engaged in general practice or specially devoting themselves to the treatment of insanity.

The intention of Dr. Steward's work being entirely practical, he abandons the ordinary systematic arrangement of the subject, and adopts the divisions into idiopathic, symptomatic, and organic insanity, as better calculated to render clearly intelligible the comparative curability of the different classes of cases.

"Pure *idiopathic insanity* is that form of the disease in which the vital and animal functions are healthily performed, the patient at the same time labouring under some particular delusion, inconsistency of conduct, or general incoherence, accompanied by altered habits and manner. Under this head will, of course, rank every case of insanity in which there is no bodily disorder or disease, whatever be the state of mind—from the first shade of disturbed intellect to total annihilation—from insanity in its mildest form to idiotcy.

"*Symptomatic insanity* I consider that form of the disease comprehending all cases, accompanied by disarrangement of the general health, evinced by a morbid state of the secretions and excretions; or, as regards any particular organ, as the uterus, by interruption or impediment of function; or when insanity is attended by an unusual state of the circulation, the heart's action being above or below the natural standard.

"By the term *organic* I mean to designate that form of insanity accompanied by disease of the brain or some other internal organ.

"This division of insanity, adapted as it is for practical purposes, leads to another, with a view to prognosis, equally important, viz. curable and incurable. First, it is to be recollected, as a fact confessedly admitted, that the curability of insanity is in an inverse ratio to the time of its duration. Upon proper and early treatment, therefore, I submit that the following forms of insanity, *there being no organic disease*, are curable, viz.:

"Cases accompanied by quickened circulation through, or congestion in, the vessels of the brain or its membranes.

"Cases accompanied by derangement of the general health, or impairment of functions.

"Cases apparently originating in excess of any kind.

"Cases accompanied by simple œdema, with general constitutional derangement.

"Cases following the sudden cessation of rheumatic pains.

"Cases following the suppression of any accustomed evacuation or secretion.

"Cases following the sudden recession of cutaneous affections.—Also probably curable, if seen within the first three months after the commencement of the attack, are—

"Cases where the delusion is limited to one subject, and the mind remains sound as regards all others, the health being undisordered."

The incurable cases are, of course, the idiopathic and the various forms of organic insanity. Predisposition must also be taken into account in the prognosis, as when this exists "few cases continue well more than two or three years at most."

Passing over the sections on the symptomatology and etiology of insanity, we shall quote from the portion of Dr. Steward's work devoted to the treatment, medical and moral. The following is Dr. Steward's opinion on the use of—

*Sedatives.* "Except in cases of insanity, occurring in persons who have been in the habit of drinking freely, and whose constitutions are shattered, and the whole frame tremulous, sedatives almost invariably do harm; so far from subduing excitement they increase it. In fact, sedatives with the insane act generally, if not invariably, as stimulants. They exercise little or no influence over the insomnia of mania, which seems, as it were, a part of the disease, which resists all remedies, and which yields only when nature, fairly tired out by long exertion, sinks exhausted, or when sleep comes, the harbinger of returning health. In what doses opium, conium, or hyoscyamus, &c., might each produce its sedative effect on the delirium of mania I know not; neither should I dare to press the medicine so far, lest its sedative effects might be fatal." (p. 59.)

This passage we cannot but regard as containing too general a condemnation of the use of sedatives in the treatment of insanity; and certainly the opinion of Dr. Steward does not accord with our own experience any more than we believe that it will with that of most other practitioners. As justly observed by Dr. Williams, the use of sedatives, like the employment of all other active remedies in these cases, requires caution. When given in cases of delirium connected with acute inflammation of the brain they are calculated to produce the evil effects ascribed to them by Dr. Steward; but administered after this inflammatory action has been subdued by antiphlogistic means, or in the cases of insanity where irritability rather than excited action exists, their use is generally highly beneficial.

*Moral treatment.* "Moral treatment may be defined the employment of means best fitted to restore the sufferer to a healthy habit of thought and action. Every experienced person knows that moral management is effective or otherwise according as its basis—classification—is well or ill chosen. The chief objects of classification ought to be security and comfort; I, therefore, found the classification I am about to suggest, not upon the different forms of insanity, but upon the self-evident principle of protection to the weak, quiet to the tranquil, comfort and security to all. With this view I divide the insane into two classes, viz *quiet* and *noisy;* and I adopt this division, first, because it includes, I think, every possible case; and, secondly, because it is of equal importance in a curative point of view, as it is essential as regards good moral management. The arrangement, to be perfect, ought to be such that the noisy cannot be heard even by each other, for a single noisy patient will generally infect the rest; and not only those generally noisy, but even those occasionally—and at that time quiet—their rest being disturbed, will become noisy. The division into quiet and noisy leads, of course, to subdivisions, viz. first into cleanly and dirty; and those again into patients *not* dangerous either to themselves or others; and patients dangerous to themselves or to others; and then again into mischievous and destructive. . . . . . For the mischievous and destructive separate galleries ought to be provided. They are, indeed, common disturbers; they incense the violent, and absolutely persecute the inoffensive; for their destructive and mischievous habits, though all in good humour, are not confined to themselves or to the property around them, but they will often destroy the clothes, &c., of their companions; besides which they are, almost without exception, disposed to appropriate to themselves whatever they can lay their hands upon. This of course leads to quarrels, if nothing worse, and ought therefore to be prevented. With respect to epileptics, some are subject to furious mania before and after a fit, are easily provoked at all times, and never quite rational. Others are only dangerous when a fit is approaching, being during the interval of convalescence in possession of their reason." [They require to be classed according to the circumstances of the case.] (p. 75.)

*Locality—Rooms and fittings—Restraint.* "No system of moral management can be good which is not founded on classification; and no classification can be perfect except when the means of accommodating all classes of patients are, *strictly speaking,* suitable. It is almost needless to suggest that the situation should be airy and cheerful, and the rooms, both day and sleeping, of ordinary size and well ventilated. This is easily accomplished as regards the orderly and convalescing, but those who most demand our sympathy and attention, who constitute our difficulties in management, who so often have been the objects of harshness and mis-treatment—are the violent, the mischievous, the helpless, and the dirty. For such cases common rooms and bedding are wholly unfitted; and unless rooms, adapted to each particular state be in readiness, neither can restraint be avoided, nor the proper means of cure or management supplied. The sleeping-rooms for these patients ought to be of convenient size and properly secure; the floor should be under-warmed, and so constructed as to admit of being readily and effectually cleansed; the bed should be such as can be removed, and another supplied in a few minutes. Of these rooms there ought to be one in every house, even though there be but a single patient. If the number exceed five, there ought to be two; and for every additional five one more; otherwise a paroxysm occurring, unjustifiable restraint cannot be dispensed with, and as restraint, *with properly warmed and properly fitted apartments,* in almost ninety-nine cases out of a hundred, is unnecessary, such arrangements ought to be enforced; for as all know, or ought to know, whatever be the present tranquillity of a patient, a change, frequently sudden, is always possible, and in most cases probable.

"I am most anxious to impress upon the minds of my readers the necessity that the floors, both of sleeping and seclusion rooms for bad patients, should be under-warmed; for as nothing but restraint will keep a patient in bed, when suffering under high excitement, it stands to reason that without under warmth he must, if at liberty, be exposed to the danger and misery of walking about, or lying, all night upon a cold, damp, and very often wet floor—boards being out of the question in these cases—should he, as constantly happens, destroy his bed or refuse to occupy it. It may, therefore, be accepted as a self-evident fact, capable of perfect demonstration, that unless the floors of at least the sleeping-rooms for bad patients be warm enough to lie and stand upon with impunity, the non-restraint system cannot be carried out with benefit or even safety to the patient. The ventilation, too, in these rooms requires to be freer than in a common room, and should be therefore of the most perfect kind.

"In a curative point of view, the construction of the rooms and beds for what are called violent patients—which are, more frequently than otherwise, curable ones—is of the utmost importance; and I firmly believe that the want of such arrangements no less conduces to the increase of incurables, than it most certainly inflicts upon the poor patients a greater and unnecessary degree of suffering.

"In commonly fitted rooms, restraint in case of violence is inevitable, and, once employed, there is no limit to its use; for, not only must it be sufficient to keep the patient in bed, but it must be so increased as to prevent him from breaking or dislocating his limbs in his efforts to free himself, and thus he remains the whole night, and often longer, the attendant being afraid to remove the restraint, lest evil to himself or the patient should follow. This continued night after night —the patient generally keeping one position—the sustaining parts soon give way, and then follow those dreadful ulcerations, so frequently terminating in the sacrifice of life." (pp. 84 et seq.)

We have now abstracted some of the more interesting and important portions of Dr. Steward's notes. His remarks, though—for the extent which his work embraces—necessarily very brief on many subjects, uniformly exhibit him to be possessed of much experience and judgment in the management of the insane.

Dr. Steward concludes his work by alluding to the necessity of some change in the law of lunacy as it at present exists, by which the anomaly shall be removed, that, while the liberty of the individual may be restrained, there exists no means of legally managing his estate, except by issuing a commission of lunacy—a step which, from the expense attending it, and the objection on the part of the friends to the publicity it entails, is usually deferred till the lunatic becomes incurable, and when after his property has sustained great injury. As the remedy of this evil, Dr. Steward suggests the delegation of a power to the commissions to enter upon the private investigation of cases reported by the friends of the patients, and to appoint provisional trustees, whose office should expire when the patient shall be deemed sufficiently recovered to reenter upon the charge of his affairs, or at a time to be fixed beforehand, or when, being deemed incurable, no objection is felt to the ordinary mode of proceeding. We think the suggestion worthy of consideration.

